# HIV Infection and the Transmission of Tuberculosis

**DOI:** 10.1093/infdis/jiu628

**Published:** 2014-11-11

**Authors:** Tom A. Yates, Ibrahim Abubakar, Frank Tanser

**Affiliations:** 1 Research Department of Infection and Population Health, University College London, United Kingdom; 2 Africa Centre for Health and Population Studies, University of KwaZulu-Natal, Mtubatuba, South Africa

To the Editor—The study by Middelkoop and colleagues [1] combining molecular and traditional epidemiology in a well-defined population is incredibly valuable. We were surprised that the index tuberculosis case patients in clusters had only twice the odds of being negative for human immunodeficiency virus (HIV), compared with other tuberculosis case patients. In the 1990s, there was little change in the incidence of tuberculosis among HIV-negative miners as the incidence in HIV-positive miners rocketed [2]. This suggests that HIV-positive persons make only a minor contribution to tuberculosis transmission.

The modest effect size in the study by Middelkoop et al [1] may be explained by the authors' definition of an index case as “the first case diagnosed within the cluster.” Given that HIV-related tuberculosis is thought to progress more rapidly to disease [3], we wonder whether many HIV-negative true index case patients might have had their tuberculosis diagnosed after HIV-positive secondary case patients.

Middelkoop et al [1] also stated that “recent transmission is responsible for the majority of tuberculosis cases in a setting where the burdens of HIV infection and tuberculosis are high. This finding was particularly marked among HIV-positive patients, including patients receiving ART [antiretroviral therapy], suggesting that ART may not provide protection against progression to tuberculosis following recent infection.” We believe that the results of the study do not support this conclusion. Were ART to provide marked protection against reactivation of tuberculosis and more modest protection against primary progression, would the same phenomenon not be observed?
